# Nonintrusive Appliance Load Monitoring: An Overview, Laboratory Test Results and Research Directions

**DOI:** 10.3390/s19163621

**Published:** 2019-08-20

**Authors:** Augustyn Wójcik, Robert Łukaszewski, Ryszard Kowalik, Wiesław Winiecki

**Affiliations:** 1 Institute of Radioelectronics and Multimedia Technologies, Warsaw University of Technology, Nowowiejska 15/19, 00-665 Warsaw, Poland; 2 Institute of Electrical Power Engineering, Warsaw University of Technology, Koszykowa 75, 00-662 Warsaw, Poland

**Keywords:** NIALM, smart home, electrical appliances, home events, load disaggregation, sensing technologies, intelligent algorithms, human behavior

## Abstract

Nonintrusive appliance load monitoring (NIALM) allows disaggregation of total electricity consumption into particular appliances in domestic or industrial environments. NIALM systems operation is based on processing of electrical signals acquired at one point of a monitored area. The main objective of this paper was to present the state-of-the-art in NIALM technologies for the smart home. This paper focuses on sensors and measurement methods. Different intelligent algorithms for processing signals have been presented. Identification accuracy for an actual set of appliances has been compared. This article depicts the architecture of a unique NIALM laboratory, presented in detail. Results of developed NIALM methods exploiting different measurement data are discussed and compared to known methods. New directions of NIALM research are proposed.

## 1. Introduction to Appliance Load Monitoring Systems

A major problem of power systems in recent years has been the constant increase in demand for electricity. In view of this problem, saving energy and reducing its consumption are promoted. Many people have joined in the initiatives aimed at increasing the energy efficiency of the places where they live. However, the users of electricity have no opportunity for conscious control of energy consumption, because their possibilities are mostly limited to analysis of accounts after settlement periods lasting usually a few months. Moreover, in both the domestic and industrial environment, dozens of appliances with different power consumptions are switched on and off several times a day. Accounts containing total energy consumption do not give any information about electricity consumption of particular appliances. Therefore, it is hard to form habits of saving energy consciously and effectively, which is one of the most important aims of smart homes.

The first purpose of developing an appliance load monitoring (ALM) system is to provide an electricity consumer with information about the energy consumption of individual appliances. This leads to limiting electricity consumption and less atmospheric pollution. Moreover, consumers with an ALM system would be aware of the appliances consuming the most energy. From a social point of view, it is important to educate people about the habit of saving energy. Obtaining information about the most energy-consuming appliances provides potential opportunities for electricity management. Some of the most energy-consuming appliances could be switched on only when there is an excess of energy in the power system. For industry, ALM systems may suggest optimal configurations of industrial machines to limit reactive power. Future possibilities of ALM systems may include diagnostics of electrical appliances [1], including monitoring of device wear (e.g., mechanical problems in rotating motors), and detection of supply network states, including dangerous inferences, voltage spikes, weakening of insulation, etc.

**Figure 1:**
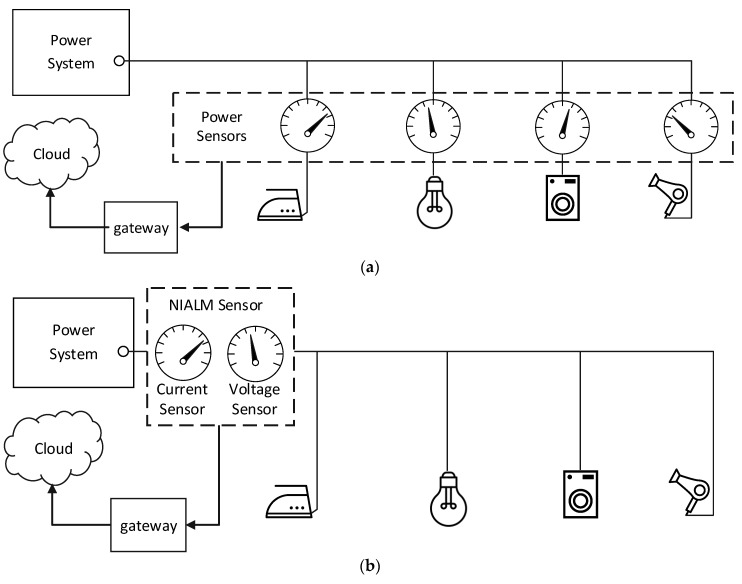
Appliance load monitoring systems: (**a**) intrusive appliance load monitoring (IALM); (**b**) nonintrusive appliance load monitoring (NIALMS).

The simplest way to monitor power consumption of all appliances is to equip them with individual electricity meters. There are many problems resulting from the fact that each monitored appliance needs an individual meter. Firstly, with the increase in the number of monitored appliances, the cost of the measuring system increases significantly [2]. Secondly, measuring data need to be collected in one central unit, which is another system element. Thirdly, data from meters need to be sent to a central unit, so electricity meters have to be equipped with a suitable interface, e.g., a radio interface. Moreover, current flowing through the monitored appliances has to flow through the meter too, which reduces the reliability of the power supply. Lastly, measuring devices consume energy. The more monitoring system elements, the more energy is consumed. Because of the mentioned arguments, such a system is called IALM—intrusive appliance load monitoring (see Figure 1a).

Another concept for solving the presented problem is a nonintrusive appliance load monitoring (NIALM) system, which also determines the energy consumption of particular appliances turning on and off in local domestic or industrial power grids. First concept of NIALM system was introduced by Hart [3], and is also known as energy disaggregation [4]. It is called nonintrusive because measurements are made solely near the energy meter, in contrast to intrusive systems where every socket or load should be equipped with a suitable sensor [5]. When new appliances are plugged into the area monitored by nonintrusive system, the hardware does not need to be expanded. Measured values are typically current and voltage of the total load [6]. A measuring system of this type is presented in Figure 1b.

Electricity is transferred in the form of voltage. Under the action of voltage, the current flows through a specific receiver. The goal of electrical signal processing algorithms is to determine individual values of currents on the basis of characteristic parameters of current, voltage, or any combination of both of these signals. Information about the operating states of individual appliances and estimations of their energy consumption are obtained by a sophisticated analysis of collected waveforms. The main challenge of NIALM is to develop methods of signal analysis which provide knowledge about the behavior of electrical appliances. This is crucial for proper system operation. The hardware of a complete NIALM system does not need to be extended with new devices in the monitored area.

**Figure 2:**
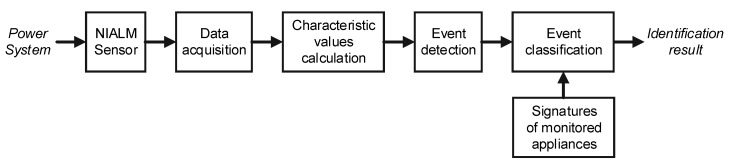
General architecture of a nonintrusive appliance load monitoring system.

The general architecture of a nonintrusive appliance load monitoring system is presented in Figure 2.

The first stage is a NIALM sensor, the main purpose of which is to transform the quantity of interest (e.g., current or voltage) into voltage with a range appropriate for the acquisition equipment. In the data acquisition stage, measuring signals are converted from analog to digital samples and saved in the memory. The sampling frequency of the recording hardware determines the type of characteristic values extracted from measured signals. Examples of characteristic values are: average power, harmonics of current, wavelet transform coefficients, root mean square (RMS) power. Characteristic value is calculated on the basis of recorded samples. The majority of methods employ more than one characteristic value. The set of characteristic values should be unique for every monitored appliance. The third stage is event detection based on analysis of the characteristic value vector. In the simplest scheme, an event is detected when the difference in two subsequent characteristic values is above the determined detection threshold. A characteristic value used often for event detection is the envelope of the current signal. Because multiple appliances work simultaneously during online operation, most methods identify only the device that has recently changed its state. The sequence of correct identifications allows which appliances were operating in the household in the particular moment of time to be determined. The characteristic values calculated after the event detection are calculated for the group of currently operating appliances, and cannot be used instantly for identification. Instead, the system also stores the set of characteristic values prior to the analyzed event. In this way, it is possible to calculate the features for the particular appliance as the difference between two vectors, calculated after the previous ***f***(*t–1*) and the last event ***f***(*t*)*:*

(1) $$f_{i}\left(t\right)=f\left(t\right)-f\left(t-1\right),$$

The event classification stage compares the calculated vector of characteristic values ***f_i_***(*t*) with the signatures of the monitored appliances. A signature is the vector of characteristic values calculated under controlled appliance operating conditions, i.e., when each appliance was turned on individually. As the result of event classification stage, the set of appliances actually operating is determined and individual energy consumption is calculated. 

It should be noted that the signal analysis methods can be varied. They take into account characteristic values calculated either when the operating state of an appliance changes, or when operating state is determined. Analyses at both mentioned types of operating state can be performed using methods belonging to one of three groups:

- Group I (fundamental 50 Hz harmonic group, LF, RMS)—analysis methods exploiting RMS of signals or the amplitude of the first current and voltage harmonic collected with a sampling frequency from fractions of Hz to several Hz.
- Group II (fundamental 50 Hz and its harmonics group, MF, harmonics)—analysis methods exploiting instantaneous values of signals (samples) collected with a sampling frequency from 1 kHz to dozens of kHz.
- Group III (high-frequency group, HF)—methods exploiting instantaneous values of signals collected with a sampling frequency from dozens of kHz to several dozen MHz.

Within each of the above groups, current and/or voltage signals are analyzed in several stages, similar to those presented in Figure 2. Analysis methods can be grouped by the duration of the analysis window. Moreover, there are methods exploiting steady-state operating states (SS) and transient states (TS) when appliance operating states change [7].

**Figure 3:**
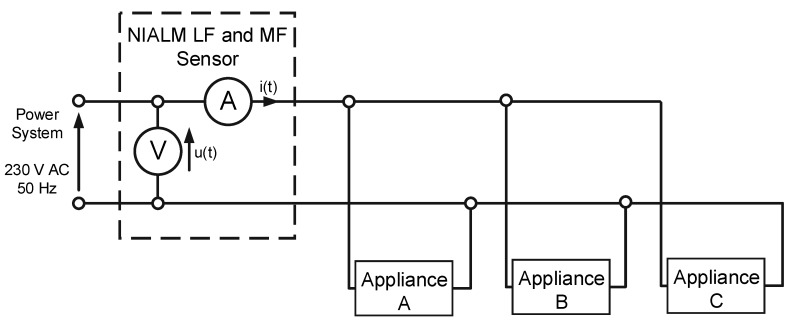
Block diagram of the measurement setup.

The aforementioned methods are described in the following sections with the use of the measurement setup shown in Figure 3. Power supply voltage and aggregate current were measured and recorded. Measured voltage was applied for a set of three appliances switched on and off apart from each other.

## 2. Group I (LF) Measurement Methods

**Figure 4:**
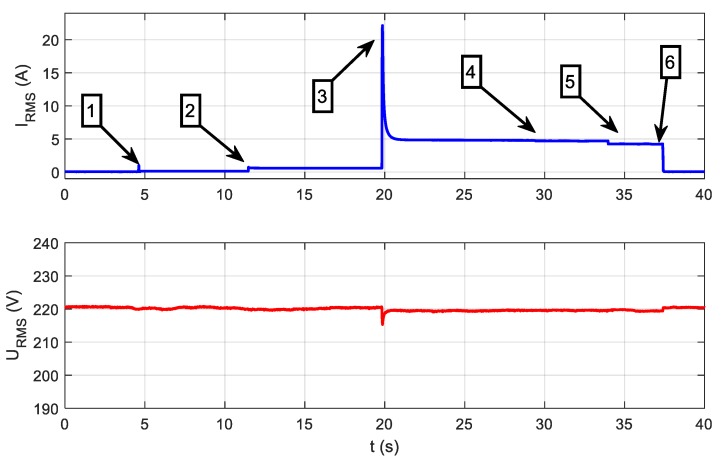
The sequence of switching on and off tested appliances.

Signals analyzed with the use of methods of group I (LF) can be evaluated using the following Equations:

(2) $$I_{RMS}=\int_{t_{0}}^{t_{0}+T}i^{2}\left(t\right)\;dt,$$

(3) $$P_{RMS}=\int_{t_{0}}^{t_{0}+T}i\left(t\right)u\left(t\right)\;dt,$$

where *i(t)* is the current signal; *u(t)* is the voltage signal; *T* is the time of analysis, which lasts one period of the fundamental harmonic or a multiple of one period; *I_RMS_* is the RMS value of the current; and *P_RMS_* is the RMS value of the power. It should be mentioned that RMS values derived from power meter are a mean value of many RMS evaluated in a 1 second or 0.5 second time interval. Signals analyzed using methods of group I are presented in Figure 4. It presents the result of measurements performed in the set of three electrical appliances detailed in Table 1, supplied from one common voltage of 230 V AC (50 Hz). 

The LED bulb (A) was switched at the moment 1. Next, at the moment 2, hair dryer (B) is switched on. Moment 3 indicates the vacuum cleaner (C) switching on. Moments 4, 5, and 6 indicate the time points at which the appliances were switched off, respectively: LED bulb, hair dryer, and vacuum cleaner.

By using the presented signals, analyses using the following methods were performed. In relation to the power grid voltage frequency of 50 or 60 Hz [8,9,10], analysis in a low-frequency range allows extraction of the parameters of the waveforms averaged over several periods. Generally, LF methods are based on analysis of power changes between steady states [11]. The most frequently used parameters of power are the average power [8,12,13,14,15,16], reactive power [17,18], and power factor [12]. The detected changes are often plotted as points in two-dimensional space. The obtained points can be assigned to groups with approximately the same changes of power. Groups with the same value but the opposite sign of change correspond to turning on and off the same appliance. In numerous publications, hidden Markov models (HMM) and their variants [19,20,21,22,23] have been applied for analyzing power changes. Baranski and Voss [11] presented an identification method based on fuzzy clustering and genetic algorithm, while a temporal mining approach was proposed in Reference [24]. The idea of representing a dataset of power values using a graph defined by a set of nodes and a weighted adjacency matrix has been introduced in References [13,25], among others. Appliances with a finite number of states are often considered finite state machines (FSM) [3,26]. The main constraint of LF methods is their inability to distinguish appliances with similar power. For this purpose, more sophisticated acquisition hardware is exploited. 

## 3. Group II (MF) Measurement Methods

**Figure 5:**
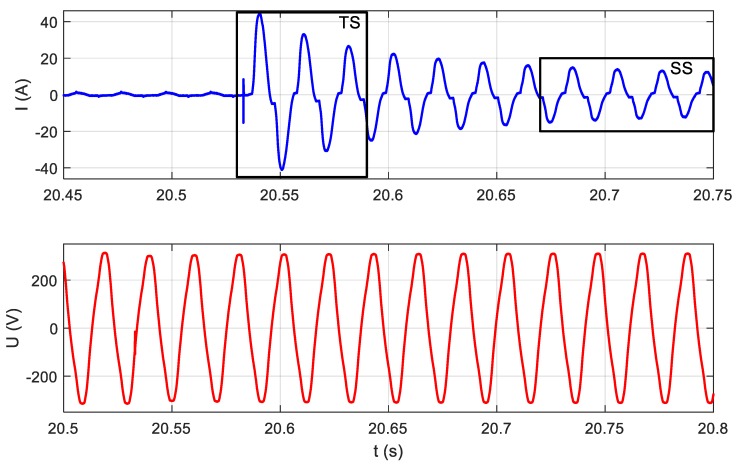
Moment 3, the vacuum cleaner switching on, in the scale of milliseconds.

**Figure 6:**
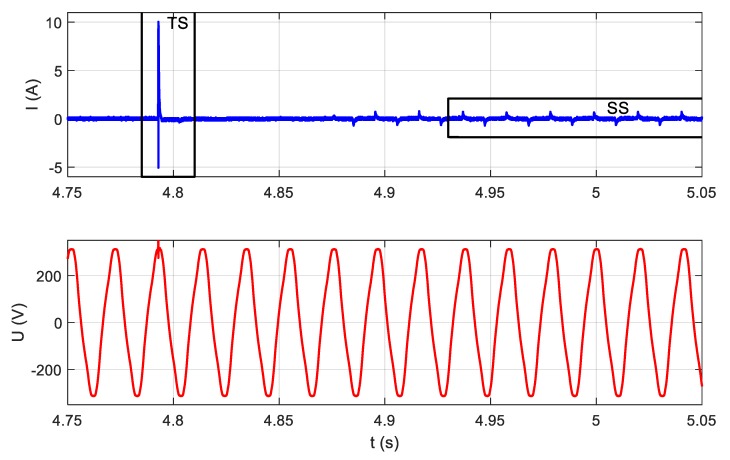
Moment 1, switching on the LED bulb (**A**).

If signals u(t) and i(t) (see Figure 3) are measured with the use of an appropriate measuring device with a sampling frequency in the range 1000 S/s to 20 kS/s, these signals will be observed as is presented in Figure 5. The waveforms presented in Figure 5 were acquired in moment 3 (see Figure 4), and the only difference is that the scale of time is more accurate. Thanks to this approach, there were visible changes in signals completely invisible for LF methods, which were particularly evident in the example of the LED bulb signals (moment 1), presented in Figure 6. LF methods are not suitable for analyzing signals that are so distorted with respect to a 50 Hz sinusoidal signal.

The majority of household appliances are nonlinear, e.g., computer or LED lamp. Hence, the current waveforms of these appliances contain significant harmonic distortion. The fact of harmonic distortion was employed in the design of NIALM methods. Parameters characterizing appliances are commonly determined on the basis of current harmonics obtained from the complex Fourier spectrum. Usually, the first 11–13 harmonics are used in the analysis, so in order to capture the microscopic parameters, it is necessary to have measurements with a sampling frequency of at least 1.5 kHz. The sample signature of an electrical appliance contains information about the amplitude [27] and the phase [28] of the subsequent current harmonics. In some works, power parameters have also been included [29]. The distribution of amplitudes and phases of individual harmonics depends on the set of appliances currently operating. The basis for identification algorithms is a comparison of signature parameters calculated from measurement data and labels of individual appliances collected during system training. Neural networks are often used as a classifier. In the input layer of the neural network, information about the harmonics of the electric current is entered, while in the output layer, the appropriate appliance category is obtained.

In Reference [30], the real and imaginary parts of the odd harmonics of the current were features characterizing devices. A number of experiments were carried out comparing the operation of three types of neural networks: radial basis function (RBF), multi-layer perceptron (MLP), and support vector machine (SVM). The presented experimental results show that average classification accuracy of developed method was about 85%. 

In Reference [31], a slightly different approach was applied. The spectrum distribution in sub-bands distributed linearly or logarithmically in the range from 0 Hz–5 kHz was calculated. Jaccard distance was used to assess the similarity of classified examples with the training data. A high identification accuracy was achieved. However, the algorithm was only tested with devices switched on individually.

A spectral envelope for estimating the power of variable speed drive (VSD) with a bulb operating in background was used in Reference [32]. The spectral envelope can also be used in the analysis of transient states. Shaw et al. [1] proposed a method of transient spectrum analysis which may be applied both in AC power grids and DC power supply in automotive applications. 

A different approach was proposed by Lang, Fung, and Lee. They proposed the taxonomy of electrical appliances based on voltage–current (V-I) trajectories [33]. In this method, graphs of current are drawn as a function of voltage. The features distinguishing devices are the shape parameters of these graphs. The authors described some features of trajectory shape which corresponded to parameters of electrical signals (e.g., the number of self-intersections as related to harmonic content). An investigation of a trajectory-based load monitoring system was presented in Reference [34]. An identification accuracy of about 75% was achieved.

## 4. Group III (HF) Measurement Methods

**Figure 7:**
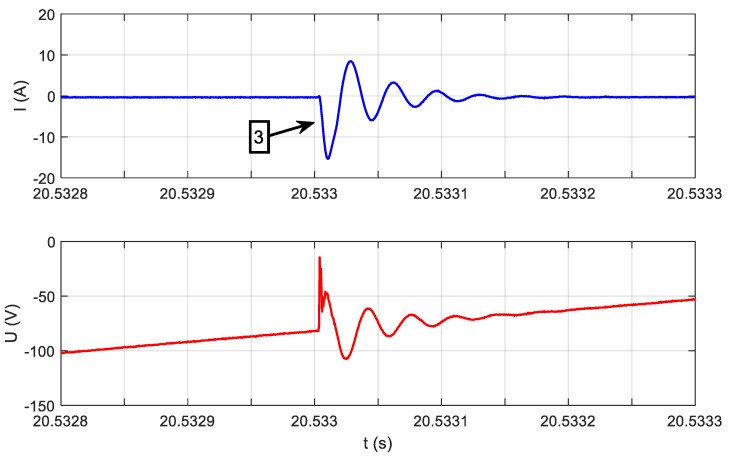
Moment 3, the vacuum cleaner switching on, in the scale of microseconds, transient state (TS).

If we look more precisely at the phenomena occurring when appliances change their state, we will see that current and voltage measured according to Figure 3 contain high-frequency (HF) components not harmonic with the 50 Hz fundamental frequency of the power supply. Information about appliances’ state changes may be included in these HF components. HF components are the response of appliance impedance to the instantaneous voltage of a power grid present at the moment of switching on an appliance. Therefore, a wide frequency band should be used for analysis. The waveforms presented in Figure 4 are expanded in Figure 7.

One group of HF methods are those characterizing transient states during switching on/off of electrical appliances. Due to the high sampling frequency of recorded data, it is necessary to apply an appropriate acquisition system that allows for initial reduction of the data, e.g., by detecting the moment of state change and recording only a significant part of the signal for further analysis [35]. Waveforms registered in transient states (TSs) very often differ significantly in shape, even if they have been measured for the same device. This makes it necessary to collect many examples of TSs, so that it might be possible to characterize them with universal and robust features. Therefore, before developing an identification system, it is necessary to prepare an appropriate database containing many TSs registered for different devices [36].

In Reference [37], a NIALM system sampling current and voltage with a frequency of 1 MHz was presented. Authors observed that TSs after switching on appliances lasted for different times, depending on the type of device. A vector of harmonics comprised from alternating real and imaginary parts of the complex Fourier transform and partial least-square regression applied to raw waveform data were recognized as the most robust features. Classification was directly related to simple statistical hypothesis testing. To improve the classification accuracy, a fusion of classifiers using both types of features was used. In the laboratory, the method allowed perfect accuracy to be obtained, but in a real household environment, a decrease of classification accuracy was observed, especially for low-power devices.

The voltage signal sampled at 5.21 MHz was used to analyze the energy consumption in Reference [38]. As the method is based solely on the analysis of transient states, the basic component (60 Hz) and its harmonics were filtered with the Notch IIR filter. Two sets of features were prepared. The first one, based on continuous wavelet transform (CWT), is a vector containing energy for selected scales and signal duration. The second feature set constitutes parameters calculated from short-term Fourier transform (STFT): averages of the individual complex Fourier spectrum components and the number of time windows. In the experiments performed, three selected appliances were tested. The STFT based method achieved an accuracy of 70%, while the use of CWT ensured 80% identification accuracy.

**Figure 8:**
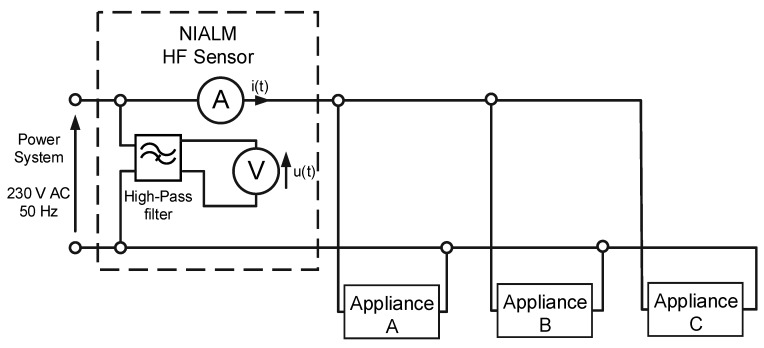
The block diagram of electromagnetic interference (EMI) measurement setup.

**Figure 9:**
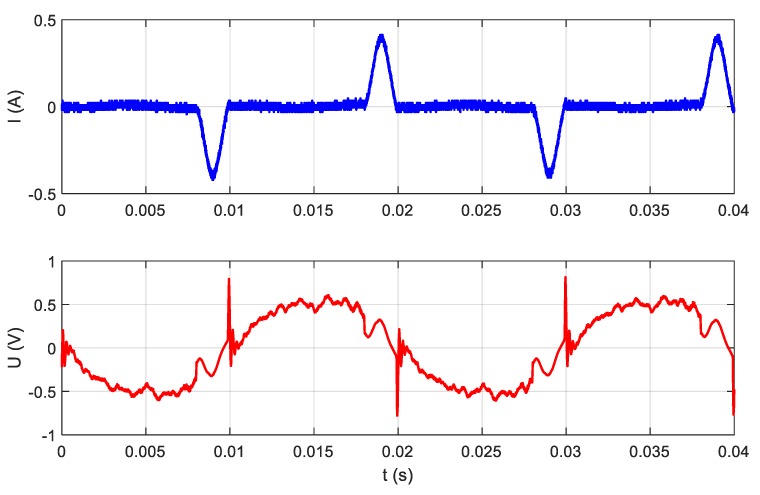
Current and high-pass (HP)-filtered voltage measured during LED bulb operation.

In most electronic appliances, switched mode power supplies (SMPS) are used. Such a solution is more effective than a classical transformer in terms of size, weight, and cost. Moreover, in the case of a transformer, the power is constant, whereas electronic appliances with SMPS get current pulses containing a large number of harmonics. Therefore, important signals contained in the high-frequency band also appear in steady states (SS). SMPS generate a high-frequency electrical noise (EMI—electromagnetic interference) during operation. The measuring system should be adjusted to extract EMI components from steady-state signals. The measurement setup from Figure 3 was modified by adding a high-pass filter in the voltage measurement path cutting of the fundamental 50 Hz frequency of the power grid voltage. It is presented in Figure 8. An example of signals measured in this measurement setup is presented in Figure 9. High-pass filter inclusion causes a phase delay of the voltage signal.

The EMI noise is specific for different electronic appliances and can be used for the identification [39]. The continuous noise is a narrowband with characteristic center frequency for different appliances. The frequency for which the level of noise is the highest is called the switching frequency and can be modeled with a normal distribution. For appliance identification, in this method, the use of switching frequency and normal distribution parameters has been proposed. These parameters are different for each appliance and are saved in an appliance library. The appliances are identified using k-Nearest Neighbor and parameters saved in the appliance library. The NIALM system that uses electromagnetic interference for appliance identification was proposed by Gupta and co-workers [39] and expanded by operating state type identification in Reference [40]. 

## 5. Extra-High-Frequency (EHF) Measurements

**Figure 10:**
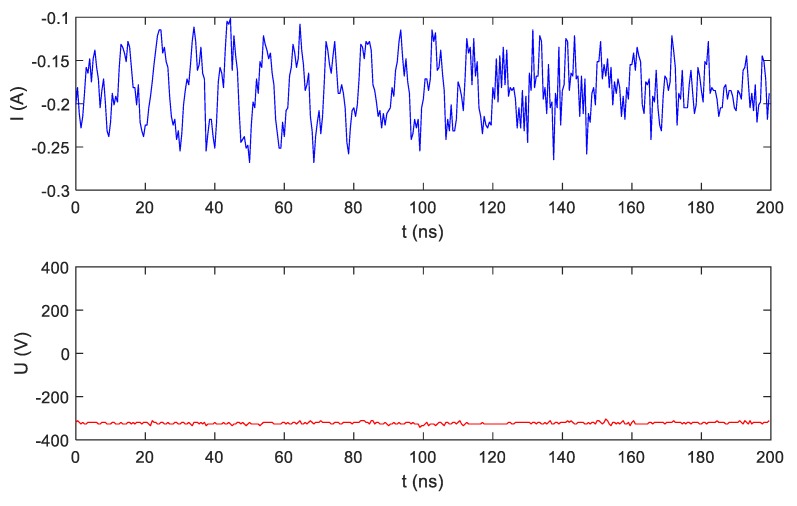
Extra-high-frequency (EHF) voltage and current recorded with frequency sampling of 2 Gs/s during LED bulb (A) operation in steady state (SS).

**Figure 11:**
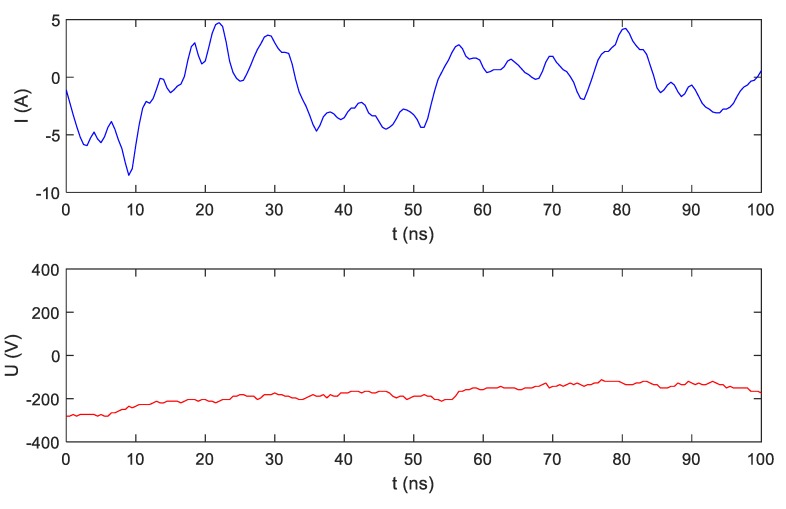
EHF voltage and current recorded with frequency sampling of 2 Gs/s during LED bulb (A) switching on (TS).

Signals measured in the measurement setup presented in Figure 3 contain components with frequencies higher than those discussed in Section 4. Other frequency components up to GHz are included in current and voltage signals. Some measurable amplitudes of extra-high-frequency (EHF) components originate from very-high-frequency (VHF) radio transmitters. It can be suspected that information characteristic for electrical appliances is included in this frequency range. So far, no one has conducted such research. Current and voltage signals were measured with a sampling frequency of 2 GHz using an Agilent InfiniiVision oscilloscope. Figure 10 presents the steady-state operation of an LED bulb (A), while Figure 11 presents a transient state during LED bulb (A) switching on. The scale of current magnitude in Figure 10 and Figure 11 is different.

It is also worth mentioning that there are alternative methods of estimating electricity consumption without measuring electrical signals. The concept of device activity detection using radio frequency interference emission was presented in Reference [41]. This method takes advantage of the fact that electrical appliances during operation generate electromagnetic fields around their enclosures. A suitable broadband antenna sensor and data acquisition system allows this disturbance to be recorded and characterized [42]. Similarly, in Reference [43], the acoustic noise produced during operation was used to identify the type of device.

## 6. Comparison of Nonintrusive Appliance Load Monitoring Methods

The accuracy of load disaggregation methods is strongly related to the number of monitored appliances. The smaller the number of devices used for the experiment, the greater the probability of a correct energy disaggregation. Similarly, a larger set of tested appliances requires more complex processing algorithms. Therefore, in order to obtain a complete overview of the state of the art in NIALM, the classification accuracy of individual appliances should be analyzed with regard to the type and number of all tested appliances in each experiment. Only this approach allows the suitability of methods in practical applications to be assessed.

Various quality measures have been used in published studies. There are various objective evaluation metrics under the umbrella of classification accuracy. The authors do not always precisely define how the accuracy of identification was determined. The quality of the load disaggregation in NIALM can be assessed on the basis of:

- accuracy of classification of events in the monitored area, where an event is a change of operating state of a particular appliance,
- accuracy of reconstructing operating states of particular appliances in every observed moment of time,
- accuracy of electricity consumption disaggregation expressed in physical quantity, e.g., in kWh, in each considered observation interval.

The most common method of accuracy evaluation in the classification issue is the confusion matrix. Confusion matrices afford the number of examples classified correctly and the number of examples assigned to an inappropriate class. In the field of NIALM, class means usually the same as appliance. Evaluation measures calculated from confusion matrices are: precision, recall, and F-measure. A detailed introduction to these measures, used widely in data mining and machine learning, is provided in Reference [44]. Application of a confusion matrix in NIALM issue was proposed in Reference [6], while in Reference [45], a study of the classifier parameters adaptation to NIALM-specific problems was provided. Depending on the processing algorithms used, evaluation metrics may be applied to the overall system performance or to the individual processing steps. Examples of evaluation metrics distinguishing event detection and classification are described in Reference [46].

It should be noted that the introduced evaluation metrics apply to the classification of detected events. The imperfection of them is that all consider only the moments of event occurrence, i.e., moments of operating state change. Under certain NIALM operating conditions, operating states may change at large intervals, e.g., at night. In this case, more important for evaluation would be the accuracy of reconstruction of the power of particular appliances in each observation interval, because one of the main goals of NIALM research is to determine which devices consume the most energy. Evaluation of NIALM by comparing the disaggregated energy consumption expressed in kWh to the total energy consumption was proposed in Reference [23]. A comprehensive approach to quality measures used in NIALM was presented in Reference [47].

Another issue is the preparation of testing data. NIALM methods are often tested initially when appliances are turned on and off individually, and no appliance is operating in the background [34]. However, this is not a sufficient testing scheme. Testing data should be aggregate signals of power, current, or voltage and current recorded during operation of tested appliances switched on/off in a certain scenario. This scenario of switching on and off appliances is often referred to as a sequence. 

Scenarios should include a variety of appliances state change combinations. This ensures that the considered method is evaluated in most possible operating conditions. Unfortunately, testing scenarios have often been collected in houses or flats, where appliances were switched on/off randomly. Moreover, appliances were often switched on/off when one or even zero appliances were operating in the background. As an example, in Reference [48], the washing machine identification was performed with high accuracy, but the figure presenting the testing sequence shows that only one appliance was operating in the background.

The mentioned issues suggest that reliable evaluation of NIALM method is demanding. Moreover, comparing different methods is more demanding. However, we have tried to determine groups of appliances which may be classified with the use of particular NIALM methods.

A literature review has been carried out in order to identify groups of appliances that can be classified in one area with the use of a given method. Due to the fundamental differences in the architecture of systems using data with different sampling frequencies, the analysis was divided into three groups: LF, MF, and HF. On the basis of the literature review, the methods used for load disaggregation in relation to the types of devices have been presented. The results are collected in Table 2 and Table 3 (LF and MF accordingly), containing, on one hand, a set of appliances monitored successfully in one area, and on the other hand, the methods used for load disaggregation listed as references to source articles. If more than one testing set was used by the authors, it was marked with consecutive letters in brackets "(a)" etc. No publication that would meet the criteria adopted during the analysis was found within the HF group.

Because the authors used different evaluation metrics, no percentage accuracy results are presented. It was only specified for each appliance whether it was possible to classify the appliance using a given method in the specific background according to authors’ conclusions. Therefore, for more information, please see the source papers. A review of the literature covered about 100 sources. Only a few of them, listed in the tables, contained evaluations of classification of particular appliances. The table legend is as follows: "+" means that appliance was in the testing set and was classified properly. "N.A." means that the particular appliance was not tested. 

The presented results show there is a lack of universal methods able to recognize dozens of any type of appliance, which is crucial in practical applications. Therefore, we still need further study in the field of NIALM.

## 7. Our Approach to Low-Frequency NIALM 

**Figure 12:**
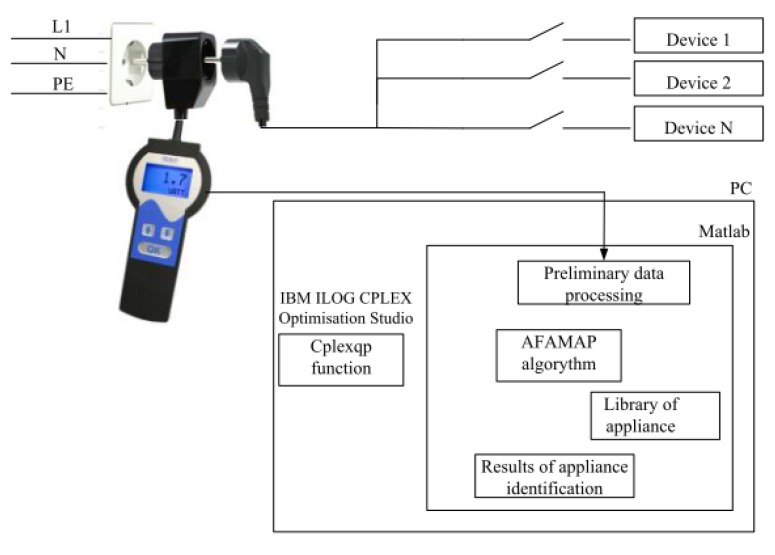
Low-frequency NIALM architecture [52].

In our first approach to NIALM systems, active power was measured with a frequency sampling of 1 Hz using a Christ CLM 1000PP ELEKTRONIK power sensor. Power samples were processed using hidden Markov models (HMM) adopted to NIALM, among others in References [19,20]. In our study, additive and differential factorial HMMs and an approximate inference algorithm (AFAMAP—additive factorial approximate MAP) were used. Cplexqp function from IBM ILOG CPLEX was used for optimization. The architecture of the proposed NIALM is presented in Figure 12. We performed experiments using many appliances. The results led to the following conclusions. Firstly, accurate identification is possible when the nominal power of the tested appliance is high enough (>40 W). Secondly, the difference in power draw of appliances must be noticeable. Examples of appliances for which this method operates properly are: electric kettle, light bulb, and microwave. We have recognized the following problems in the LF NIALM approach:

- low-power appliances, e.g., energy-saving light bulbs, were not recognized properly,
- the system was confused when two appliances with similar nominal power were tested,
- when variable power load was operating in the background, identification was inaccurate.
- appliances with variable power, e.g., washing machine, induced false event detection as the result of rough current and power draw.

For the reasons presented, we decided to design a universal laboratory that would be able to perform experiments with methods exploiting both current and voltage signals sampled with frequency modifiable in wide range.

## 8. Architecture of the NIALM Laboratory

As a part of our research, the NIALM laboratory was created. The creation was a process that took more than a year, and required a couple of different approaches, starting from the use of digital fault recorders (DFR) normally utilized in high-voltage networks for recording of currents and voltages appearing during various kinds of disturbances. At the beginning of our research, dual speed DFR (BEN6000) was used. It allows recording of instantaneous values of current and voltage signals with a sample rate up to 12 kHz. RMS values of currents, voltages, and their derivatives (active power, reactive power, power factor etc.) were recorded with a speed of 50 samples per second. Numerous tests performed led us to conclusion that there was a need for other types of recorders that would be able to record samples with frequencies of tenths of kHz and few MHz simultaneously. Therefore, we developed two recorders (NIALMREC), one (MF) with a sample rate up to a few hundred kHz, and the second (HF) operating with frequencies up to 10 MHz. Both devices, based on PC, DAQ cards, and the LabVIEW program, work according to our wishes. To have the possibility of performing tests in many locations, we decided to set up a complete measuring system consisting of two recorders in one movable 19 inch rack, equipped with many electrical sockets for plugging in household appliances. 

Moreover, we also decided to have the same power supply environment and the same set of devices for testing purposes. Because of mentioned reasons, we set up our laboratory in a dedicated room located in Warsaw University of Technology, where the new supply circuits (electrical installation) were made according to our requirements. For the purpose of this stationary laboratory, the second measuring system, composed of two recorders (data acquisition hardware), was made and installed in a 19 inch rack. As the result, the laboratory consisted of both demonstrator and measurement system functions to verify the designed system model and automatic identification algorithms contained therein.

### 8.1. Electrical Installation

**Figure 13:**
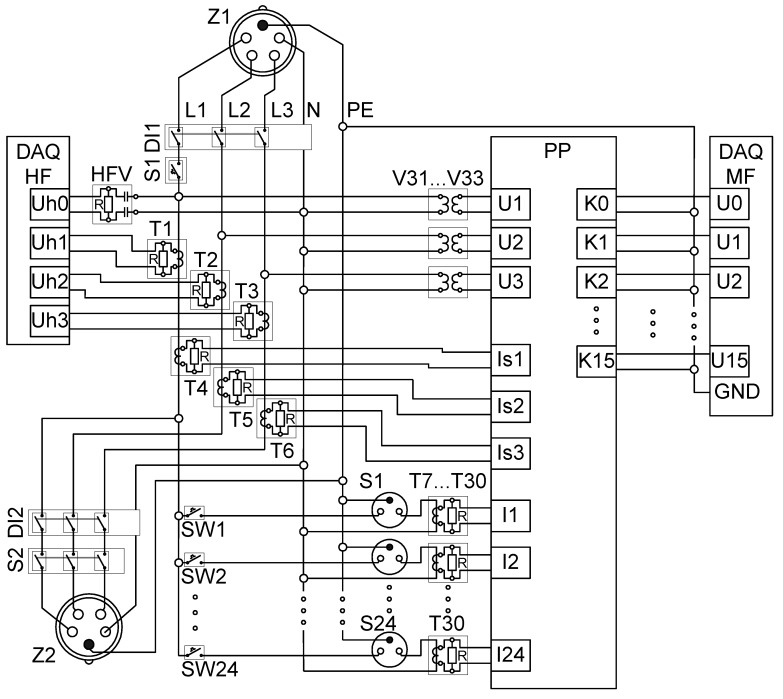
Architecture of the NIALM laboratory.

The architecture of the electrical installation is presented in Figure 13. Power supply in the form of three phase voltages (3 × 230 V/400 V), N and PE was connected to socket Z1. The main differential protection relay DI1 was then installed. One phase voltage (L1) was connected through switch S1 to the main line, supplying 24 single phase sockets (S1 to S24) via 24 dedicated switches (SW1 to SW24). Each Sx socket also had connections to N and PE. The current transducer model SCT-013-020 was used as a current sensor (T7 to T30). In serial to each Sx socket, one current sensor Tx allowing individual measurement of current was connected. In order to measure the summary currents in each phase, three current sensors (T4 to T6)—current transducer model SCT-013-020—were connected in serial to the main supply lines L1, L2, and L3. The MF measuring system could also measure three phase voltages using voltage sensors V31 to V33. The MF system also used a very flexible solution of measuring the channel switching matrix (PP) utilizing an Ethernet network STP (shielded twisted pair) patch panel, to which all voltages from the current sensors (signals: Is1, Is2, Is3, I1 to I24) and voltage sensors (signals: U1, U2, U3) were connected in the form of F/UTP cables. In each of those cables, only one twisted pair of wires (among four) was used to transfer the signal. The signals were then connected to the desired channels (U0 to U15) of the middle-frequency data acquisition system (DAQ MF) using an STP patch cord. 

The HF measuring system consisted of dedicated current (T1, T2, T3) and voltage (HFV) sensors. Sensors’ output voltage signals were connected by coaxial cables to the inputs of the high-frequency data acquisition system (DAQ HF).

### 8.2. Data Acquisition Hardware

**Figure 14:**
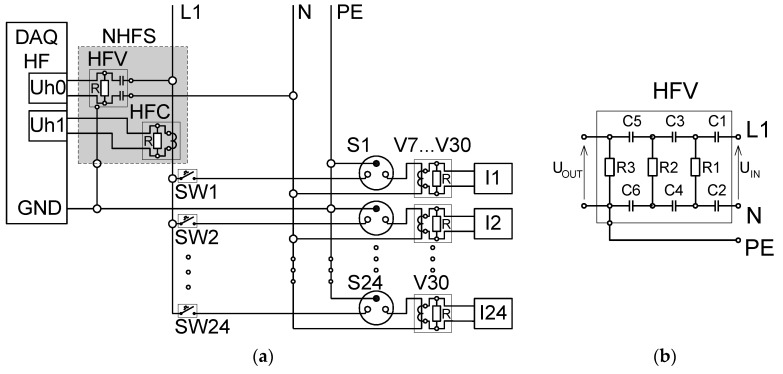
(**a**) Schematic diagram of the high-frequency voltage and current measurement setup; (**b**) schematic diagram of NIALM high-frequency voltage sensor (HFV).

One of the assumptions made while developing the laboratory was to provide the possibility of testing methods based on medium-frequency (MF) and high-frequency (HF) measurements. For this reason, the data acquisition was performed in two paths using two PCs class Intel i5. The computers were equipped with fast SSDs for online data processing and large capacity HDDs for data storage. One computer was equipped with a 16 bit Advantech PCIE-1816H card, sampling in 16 channels up to 65 kS/s for MF measurements. In the second one, a 12 bit Advantech PCIE-1744AE card was installed. This Advantech DAQ card allowed sampling with frequencies up to 10 MS/s on two channels simultaneously, or up to 20 MS/s on a single channel for HF measurements. The NIALM high-frequency measurement setup equipped with a NIALM high-frequency sensor (NHFS) is presented in Figure 14a. Following the measurement setup presented in Reference [39], a high-pass filter (Figure 14b) was installed in the HF voltage measurement path to eliminate the fundamental component of high-amplitude voltage when steady-state (SS) characteristics of appliances are extracted. It should be noted that the PC case via the power outlet was connected to a protective conductor (PE). Consequently, the acquisition card connector armor providing the measurement signal was also connected to the protective conductor (Figure 14a). The result is that one of the branches of the filter was always shorted. The cut-off filter frequency in this system was 25 kHz. The filter consisted of three CR stages (Figure 14b). The values of elements were, accordingly: C1, C2, C3, C4, C5, C6 = 100 nF, R1 = 90 Ω, R2 = 600 Ω, R3 = 1000 Ω. In order to ensure the safety of the measurement system, high-voltage capacitors were used. For measurements of transient states (TS), the filter was removed.

### 8.3. Software Supporting Measurement Layer

Software supporting the acquisition cards was implemented in the LabVIEW environment. The basic function of the software was to save to the file buffers all samples obtained during acquisition from the current and voltage sensors. In the case of the MF card, it was possible to simultaneously verify the identification algorithms based on the energy consumption calculated from the current measurements for individual devices. The software included the following functions:

- Simultaneous acquisition of measurement data from at least two input channels configured to measure voltage and current,
- Presenting the waveform of the signal from several channels (virtual oscilloscope),
- Presenting Fourier spectra of two measured signals,
- The ability to adjust FFT analysis parameters,
- Writing to files all observed samples of signals as well as calculated spectra.

**Figure 15:**
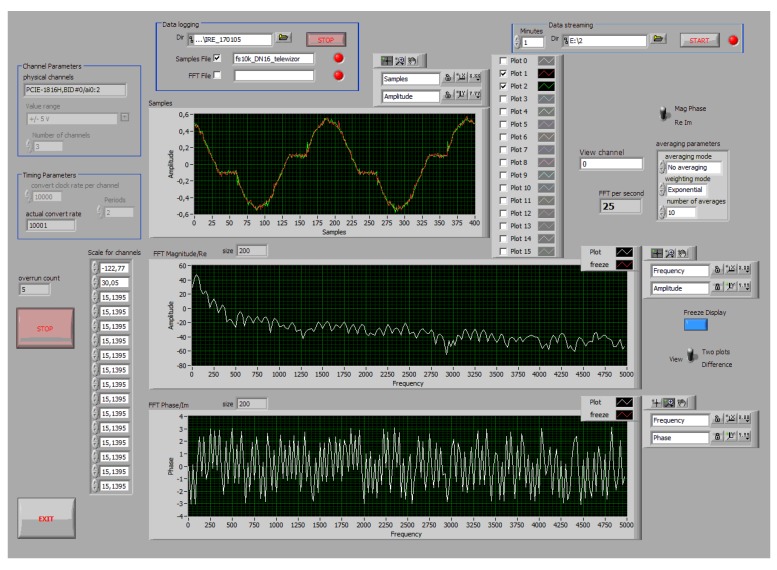
Main window of data acquisition software.

The main window of the prepared software is presented in Figure 15.

## 9. Medium-Frequency Characteristic Values

**Figure 16:**
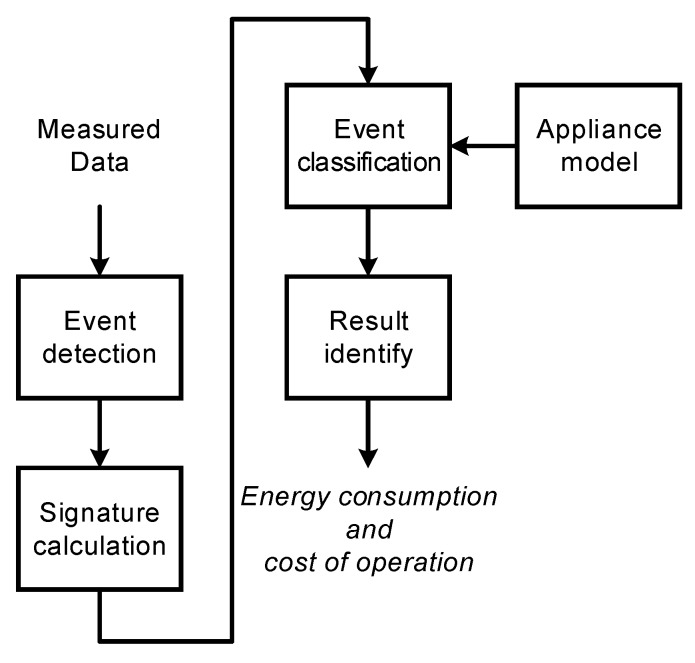
The proposed NIALM architecture.

Using the prepared measurement setup, we were able to verify and develop the implemented algorithms for data processing and identification in an environment similar to a real place in which a NIALM system might be installed. In our NIALM system [Patent 1], presented in Figure 16, voltage and current were recorded with frequency sampling of 2 kHz. 

Appliance models were developed during the training stage, when devices are turned on individually. Two waveforms representing 500 ms of current and voltage signals were processed to obtain a vector of parameters constituting the pattern (label) of an appliance. For better characterization of appliances, patterns were calculated one hundred times for each appliance. Therefore, the appliance model consists of many patterns obtained in similar operating conditions. During the experiments, we observed that device models based only on current and power parameters did not describe unambiguously electrical appliances, especially if the same power supply circuit supplied a number of different appliances. If we exploit only the AC current to determine characteristics of a device, we assume that the grid is an ideal voltage source, what is not truthful. Therefore, it was necessary to apply a method for determining parameters of electrical equipment which were not the features of the current flowing through it, because the current value depends on the supply voltage. Finally, we decided to use the following pattern, consisting of 133 parameters calculated on the basis of measured voltage and current signals (the brackets contain their numbers):

- Amplitudes of the first 16 harmonic components of the current (50 Hz, 100 Hz, etc.) (1–16),
- Phase shifts of the first 16 harmonic components of the current (17–32),
- Root mean square (RMS) of the current (33),
- Mean value of the current (34),
- Maximum current value (35),
- DC component in the current (36),
- Mean power (37),
- Values of the active power in the first 16 harmonics (38–53),
- Values of the reactive power in the first 16 harmonics (54–69),
- The first 16 harmonics of the real current components (70–85),
- The first 16 harmonics of the imaginary current components (86–101),
- The first 16 harmonics of conductances (102–117),
- The first 16 harmonics of susceptances (118–133).

**Figure 17:**
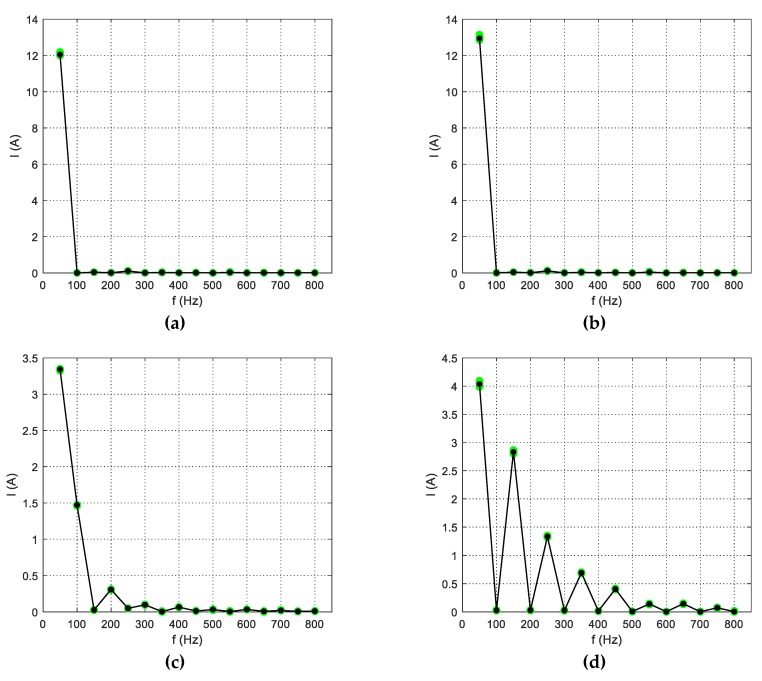
Current harmonic components (1–16) of (**a**) electric kettle; (**b**) iron; (**c**) hairdryer; (**d**) vacuum cleaner.

**Figure 18:**
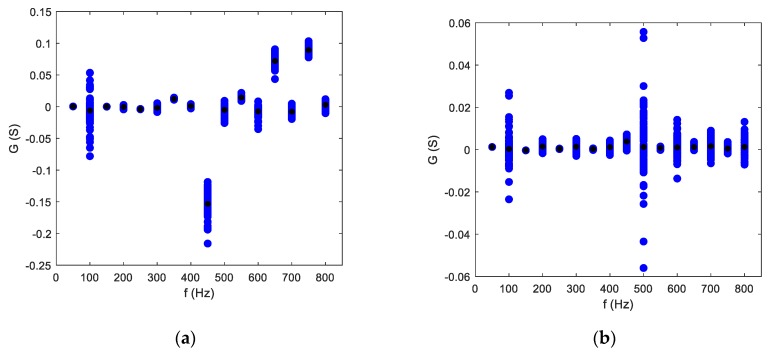
Conductance harmonics (102–117) of (**a**) LED bulb; (**b**) incandescent bulb.

Selected features of some appliances are presented in Figure 17 and Figure 18. Some appliances, like the hairdryer or vacuum cleaner, had unique harmonic content, allowing characteristic parameters to be distinguished even visually, while appliances with similar power and impedance like the electric kettle (2200 W) and iron (2200 W) differed slightly in the magnitude of the first current harmonic only (50 Hz). Another interesting issue was the content of conductance harmonics. Presented in Figure 18b, the graph for the incandescent lamp had approximately constant magnitudes of conductance harmonics because resistance content in its impedance was dominant. What is worth emphasizing is that the LED bulb had a visible increase in the conductance amplitude for higher harmonics, and, moreover, a negative magnitude of the 450 Hz harmonic. This negative conductance indicates that at this frequency, the LED bulb generates energy [Patent 2]. This indicator can be used also in recognizing appliances generating higher harmonics, and hence worsening quality parameters of the power supply voltage and thus electric energy quality.

**Figure 19:**
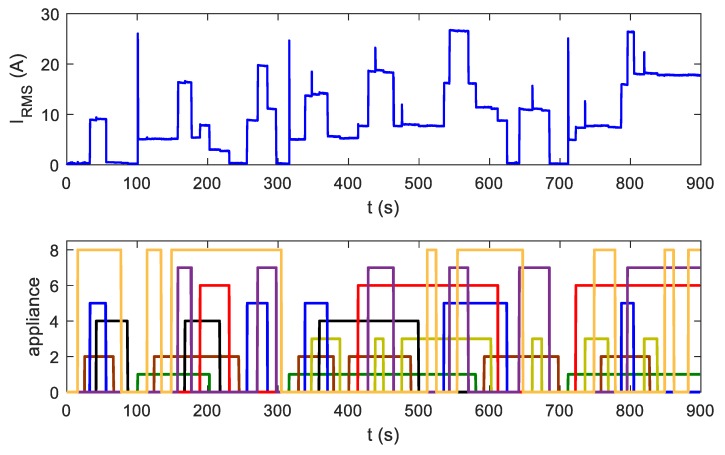
Testing sequences of appliance operation. Different colors in the bottom refer to different appliances.

Besides acquisition data for particular devices, we also recorded sequences of appliance operation. Overall current and voltage signals during the operation of multiple appliances switched on and off in different combinations imitated events in the real environment of system installation. Information about device states at specific times were attached to waveforms. This allowed verification of the correct operation of the identification algorithms. Sequences lasted from 5 to 100 min. Figure 19 presents an example of such a sequence.

## 10. Identification Results—MF Methods

The sets of recorded and processed data described above enabled us to perform a number of experiments aimed at verifying the quality of prepared patterns. We tested several artificial intelligence algorithms, adjusting their parameters in such a way as to obtain the best results for appliance identification. Moreover, we prepared a complete online NIALM system able to perform disaggregation automatically. Below, we briefly present the results of some experiments. In all experiments, the same six two state devices were considered: power-saving lightbulb, dryer, vacuum cleaner, mixer, juicer, and kettle. Table 4 lists the identification results for these experiments. 

In experiments A and B, because of the feature additive criterion described in Reference [46], among others, non-additive features designed previously were removed from the set of features used for algorithm testing. Experiments C and D were performed to adjust the optimal parameters of Random Forest and Rule Induction algorithms [57]. Results listed in [ 4 were reached when Random Forest was trained with 13 trees, while optimal training configuration for the Rule Induction algorithm was obtained for α = 10 and β = 0.05. The number of rules was equal to 21. Because there was no knowledge about parameter significance for the device identification, in experiment E, feature selection methods limiting the number of parameters to important ones only were applied. Therefore, two algorithms were exploited: one for reducing the set of features and the second for verifying the reduction efficiency. The classification accuracy was expected to be the same for both cases, but with a shorter training time for the second set. The applied Decision Tree classifier achieved an accuracy of 92.57% for the optimal set of features. In experiment F, a fusion of three classification algorithms was implemented. Fusion of three classifiers (Decision Tree, Rules Induction, and Random Forest) minimized the threat of the decision uncertainty when outcomes of the subsequent modules were equally distributed among different appliance identifiers. The overall ensemble achieved an overall accuracy of 92.96%.

## 11. High-Frequency Experiments

**Figure 20:**
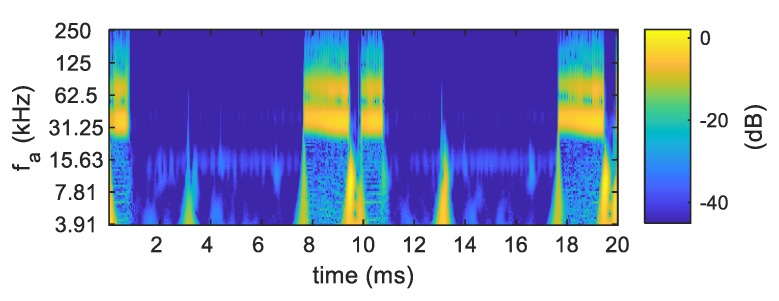
Scalogram of voltage signal during fluorescent lamp (CFL) and pedestal fan operation.

The genesis of our experiments was the research presented in Reference [39]. The aim of the experiments was to record high-frequency components of a voltage signal, which are probably not the harmonics of the 50 Hz power supply network frequency, but electromagnetic inference generated by electrical appliances. We repeated the experiment carried out by American team using the measurement method proposed in their paper [39]. Although the fundamental harmonic of the voltage signal was filtered by analog high-pass filter, we were not able to perceive high-frequency voltage components characteristic for every tested appliance. We observed distinctive amplitudes at some frequencies only during operation of a few devices (e.g., an old type of LCD monitor). Components of all other devices were invisible because of high level of the high-frequency signals in the background. Therefore, we modified the measurement setup by introducing inductance into the electricity transmission line in order to separate interferences generated by other appliances powered by the same low-voltage grid. Houses in USA are powered from a single transformer, while, in a European low-voltage grid, one transformer supplies appliances in several houses, flats etc. Modification of measurement setup decreased the level of emissions in the background, enabling us to observe differences in the spectra obtained for different devices. Signals were processed using Wavelet Transform. An example of the obtained results is presented in Figure 20.

The conclusion drawn from the performed experiments was that high-frequency electromagnetic inferences generated by appliances may contain components characteristic for some appliances. Unfortunately, in a European power grid, it was impossible to observe them because of the high level of emissions in the background. Therefore, we conclude that the actual challenge is to detect and identify operation of low-power appliances like energy-saving lamps, LEDs, or switched-mode power suppliers.

Due to the limited volume of the article, we were not able to include the results of further experiments based on high-frequency signals recorded during electrical appliance operation. We developed methods of signal processing allowing different types of appliances to be distinguished using both SS high-frequency components [Patent 3] and TS high-frequency components [Patent 4] of voltage or current signals. Acquisition and processing of signals during appliance switching on allows precise characterization of some appliances, because the amplitudes of transient signals are significant. However, steady states may be also a source of high-frequency features characterizing appliances, provided that the time of occurrence of the high-frequency oscillation is not lost. In both cases, it is necessary to perform signal analysis at a particular moment of time. That enforces the application of methods other than the previously applied time–frequency analysis. The introduced topic, along with a description of the measurement method and examples of our analysis results, will be presented in our next article.

## 12. Conclusions

The NIALM system may be an important instrument allowing improvement of effective use of energy by particular consumers. The NIALM system solutions proposed so far do not allow all types of devices to be identified in all operating conditions. In particular, there is a lack of experimental results in which the accuracy of particular appliances was evaluated. In our research, a multilateral approach [56] analyzing data obtained using various measurement setups was applied. We prepared a comprehensive NIALM laboratory adapted to perform various experiments. Tests based on a set of over 100 features and many methods of artificial intelligence were conducted. We achieved identification results similar to those presented by other authors. The performed experiments showed that the use of frequency analysis of EMI interference in appliance identification devices cannot be implemented in the European power grid. 

We also analyzed high-frequency signals that appear in both steady and in transient states. The methods of time–frequency analysis that we will present in the next paper will show new features of devices that can be used to characterize electrical appliances. We have developed a method that uses high-frequency waveforms caused by introducing an artificial energy receiver for a short time period, in order to cause disturbance and measure the system’s response to this disturbance. On the basis of this response, we can specify which appliance was currently switched on.

## 13. Patents

*Patent 1:*  *Kowalik R., Winiecki, W., Januszewski M., Nogal Ł., Łukaszewski R., Bilski P., Bobiński P., "Method and the device for identification of electrical energy receivers", PL patent no. PAT.229626, 12.03.2018.*
*Patent 2:*  *Winiecki W., Kowalik R., Łukaszewski R., Bilski P., Januszewski, M., Nogal Ł., Wójcik A., "Method and the system for identification of a source of harmonic distortions of power network, preferably caused by energy receivers", PL patent no. PAT.229627, 12.03.2018*
*Patent 3:*  *Kowalik R., Winiecki W., Dowalla K., Łukaszewski R., Wójcik A., Bilski P., Nogal Ł., "Device for the identification of the electrical appliances in the power grid and the method of the appliances identification in the power grid", PL patent no. PAT.232306, 6.02.2019.*
*Patent 4:*  *Winiecki W., Kowalik R., Wójcik A., Łukaszewski R., Dowalla K., Bilski P., "Device for detecting changes of the operation mode and identification of appliances in the power grid and the method for detecting changes in the operation mode of appliances in the power grid", PL patent no. PAT.232305, 4.02.2019*

## Figures and Tables

**Table 1:** Tested appliances.

#	Name	Producer	Type	Max Power
A	LED bulb	Osram	AB30526	17 W
B	Hair dryer	Apollo	SUA-2000-SIL	2000 W
C	Vacuum cleaner	Zelmer	ZVC425HT	1000 W

**Table 2:** Groups of appliances possible to classify properly using selected low-frequency (LF) methods.

Source Appliance	[49] (a)	[49] (b)	[21]	[50] (a)	[50] (b)	[50] (c)	[15]	[25] (a)	[25] (b)	[25] (c)	[25] (d)	[25] (e)	[51]	[19]	Our Study [52]
Air conditioner	+	N.A.	N.A.	N.A.	N.A.	N.A.	N.A.	N.A.	N.A.	+	N.A.	N.A.	N.A.	N.A.	N.A.
Bath outlet	N.A.	N.A.	N.A.	N.A.	N.A.	N.A.	N.A.	N.A.	N.A.	N.A.	N.A.	N.A.	N.A.	+	N.A.
Bulb	N.A.	N.A.	N.A.	N.A.	N.A.	N.A.	N.A.	N.A.	N.A.	N.A.	N.A.	N.A.	N.A.	N.A.	+
Dishwasher	N.A.	+	+	N.A.	+	+	+	N.A.	+	N.A.	+	N.A.	+	N.A.	N.A.
Dryer	N.A.	N.A.	N.A.	+	+	+	N.A.	N.A.	N.A.	N.A.	N.A.	N.A.	N.A.	N.A.	N.A.
Electronics	N.A.	N.A.	N.A.	N.A.	N.A.	N.A.	N.A.	N.A.	N.A.	+	N.A.	N.A.	N.A.	N.A.	N.A.
Fridge	N.A.	N.A.	+	N.A.	+	+	N.A.	+	+	+	N.A.	+	+	N.A.	N.A.
Fan	N.A.	N.A.	N.A.	+	N.A.	N.A.	N.A.	N.A.	N.A.	N.A.	N.A.	N.A.	N.A.	N.A.	N.A.
Furnace	+	N.A.	N.A.	N.A.	N.A.	+	+	N.A.	N.A.	N.A.	N.A.	N.A.	N.A.	+	N.A.
Freezer	N.A.	N.A.	N.A.	N.A.	N.A.	N.A.	N.A.	N.A.	N.A.	N.A.	+	+	N.A.	N.A.	N.A.
Heater	N.A.	N.A.	N.A.	+	N.A.	N.A.	N.A.	N.A.	N.A.	N.A.	N.A.	N.A.	N.A.	N.A.	N.A.
Iron	N.A.	N.A.	N.A.	N.A.	N.A.	N.A.	+	N.A.	N.A.	N.A.	N.A.	N.A.	N.A.	N.A.	N.A.
Kettle	N.A.	N.A.	+	N.A.	N.A.	N.A.	N.A.	N.A.	N.A.	N.A.	+	+	N.A.	N.A.	+
Kitchen outlet	N.A.	N.A.	N.A.	N.A.	N.A.	N.A.	N.A.	N.A.	+	+	N.A.	N.A.	+	N.A.	N.A.
Lighting	N.A.	+	N.A.	N.A.	N.A.	N.A.	N.A.	N.A.	N.A.	N.A.	N.A.	N.A.	N.A.	N.A.	N.A.
Microwave	N.A.	N.A.	+	N.A.	+	+	N.A.	+	+	+	+	+	+	+	+
Oven	N.A.	N.A.	N.A.	N.A.	+	N.A.	+	N.A.	N.A.	N.A.	N.A.	N.A.	+	N.A.	N.A.
Stove	N.A.	N.A.	N.A.	N.A.	N.A.	N.A.	+	N.A.	+	+	N.A.	N.A.	N.A.	N.A.	N.A.
Washing Machine (washer-dryer)	N.A.	+	N.A.	N.A.	N.A.	N.A.	+	+	N.A.	N.A.	+	+	+	+	N.A.
*Number of +*	2	3	4	3	5	5	6	3	5	6	5	5	6	4	3

**Table 3:** Groups of appliances possible to classify properly using selected medium-frequency (MF) methods.

Source Appliance	[53]	[48]	[54]	[55]	Our Study [56]
CFL (compact fluorescent lamp)	N.A.	N.A.	+	N.A.	+
Charger	+	N.A.	N.A.	N.A.	N.A.
Electronic	N.A.	+	N.A.	N.A.	N.A.
Fan	N.A.	N.A.	+	+	N.A.
Fluorescent light	+	N.A.	N.A.	N.A.	N.A.
Furnace	N.A.	+	N.A.	N.A.	N.A.
Hairdryer	N.A.	N.A.	N.A.	N.A.	+
Heater	N.A.	N.A.	+	N.A.	N.A.
Incandescent light	+	N.A.	N.A.	+	+
Iron	N.A.	N.A.	+	N.A.	+
Kettle	N.A.	N.A.	N.A.	+	+
Kitchen outlet	N.A.	+	N.A.	N.A.	N.A.
Laptop	N.A.	N.A.	+	N.A.	N.A.
LED	N.A.	N.A.	N.A.	N.A.	+
Microwave	N.A.	+	N.A.	N.A.	N.A.
PC	+	N.A.	N.A.	+	N.A.
Vacuum cleaner	N.A.	N.A.	N.A.	N.A.	+
Washing Machine	N.A.	+	N.A.	N.A.	N.A.
Number of +	4	5	5	4	7

**Table 4:** Overall identification accuracy for several algorithms.

Experiment	Identification Algorithm	Number of Events	Applied Features	Identification Accuracy
A	kNN	128	34, 36–133	85.69%
B	Random Forest	128	34, 36–133	85.69%
C	Rule Induction	84	1–69	65.47%
D	Random Forest	84	1–69	87.69%
E	Decision Tree	175	34, 36–39, 46, 49, 55, 56, 60, 70, 71, 74, 77, 79, 80, 87, 88, 92, 94	92.57%
F	Ensemble (DT, RI, RF)	128	1–69	92.96%
